# Brain Abscess as a Complication of Hereditary Hemorrhagic Telangiectasia: A Case Report

**DOI:** 10.7759/cureus.35572

**Published:** 2023-02-28

**Authors:** Syed Muhammad Hussain Zaidi, Masharib Bashar, Muhammad Saad Choudhry, Shehzeen F Memon, Shahzeb A Memon

**Affiliations:** 1 Internal Medicine, Dow University of Health Sciences, Civil Hospital Karachi, Karachi, PAK; 2 Internal Medicine, University of Toledo, Toledo, USA; 3 General Surgery, Dow University of Health Sciences, Civil Hospital Karachi, Karachi, PAK; 4 Surgery, Dow University of Health Sciences, Civil Hospital Karachi, Karachi, PAK

**Keywords:** arteriovenous malformations, osler-weber-rendu syndrome, pulmonary avm, hereditary hemorrhagic telangiectasia, brain abscess

## Abstract

An 18-year-old male, previously diagnosed with hereditary hemorrhagic telangiectasia (HHT), presented to the outpatient department with a complaint of generalized seizures and fever for the past five days. He had a history of recurrent epistaxis, progressive shortness of breath, and cyanosis. Magnetic resonance imaging (MRI) of the brain revealed an abscess in the temporoparietal region. A computed angiogram of the pulmonary vasculature showed the presence of arteriovenous malformation (AVM). A four-weekly antibiotic regimen was initiated, which resulted in a profound improvement in symptoms. A brain abscess can arise as a complication of vascular malformation in a patient with HHT, providing a nidus for bacteria to migrate toward the brain. Early recognition of HHT is essential in these patients and their affected family members, as screening can help us prevent complications at an earlier stage.

## Introduction

Hereditary hemorrhagic telangiectasias (HHT), also known as Osler-Weber-Rendu syndrome, is an autosomal dominant genetic disorder characterized by arteriovenous malformations (AVMs). AVMs are found predominantly in the lungs (50%), liver (30-70%), brain (10%), and a very small proportion (less than 1%) in the spinal cord [1]. The criteria commonly used for the diagnosis of AVM in HHT are called the Curaçao criteria, which include spontaneous recurrent epistaxis, multiple telangiectasias in typical locations, proven visceral AVMs (lung, liver, brain, and spine), and a first-degree relative with HHT. If three or all four of the above-mentioned criteria are met, a patient is labeled with a "definite HHT" diagnosis while two indicate "possible HHT" [2]. About 70-80% of lung AVMs are formed due to HHT [3]. Bleeding from lung AVMs is relatively uncommon, but, if it occurs, can cause hemoptysis or hemothorax. Large vascular malformations in the lung allow deoxygenated blood from the right ventricle to bypass the alveoli, causing cyanosis, dyspnea, clubbing, or cerebral abscess in the patient [4]. Abscesses can also develop in other organs, as microorganisms can bypass the pulmonary vein and enter the systemic arteries [4].

## Case presentation

An 18-year-old male presented to the outpatient department with complaints of high-grade fever, generalized tonic-clonic seizures, and dry cough for the last five days. The fever was continuous in nature with rigors and chills. The cough was non-productive and occurred in bouts. There was no associated history of rash, joint pain, oral ulcers, abdominal pain, chest pain, hemoptysis, or any specific urinary complaint. The episodes of seizures usually self-resolved in 20 to 30 seconds, and some of them were associated with frothing. Past medical history revealed a diagnosis of hereditary hemorrhagic telangiectasia (HHT) two years back. The diagnosis was made based on a prolonged history of recurrent atraumatic nosebleeds for the past 10 years that were self-limiting. These episodes of epistaxis occurred at variable intervals. They were not associated with bleeding from any other site or orifice, easy bruising, bleeding gums, hemoptysis, or hemarthrosis. Furthermore, for the last five years, he was also experiencing shortness of breath on exertion. Shortness of breath was progressive, continuous, and occurred on walking around 100 meters but subsided with rest. It was not associated with hemoptysis, orthopnea, pedal edema, or abdominal distention. However, his parents noticed cyanosis of the fingers, lips, and tongue, which became permanent for the last six months. A similar pattern of symptoms was also present in the immediate and paternal family. On general physical examination, the patient was of average height and lean built, responsive, and oriented to time, place, and person. The patient was vitally stable except for a fever of 103 Fahrenheit. Anemia, odontogenic infection, jaundice, and edema were not observed. The jugular venous pressure was raised to about 9 cm and the lymph nodes were not palpable. His oxygen saturation (SaO2) was revealed to be 88%. He had florid central cyanosis visible on the tongue, lips, and face as well as on the hands along with prominent clubbing. On cardiac examination, the apex beat heaved in character in the fifth intercostal space in the midclavicular line, 9 cm from the sternal border. No parasternal heave or murmur was appreciable, but there were epigastric pulsations, loud S1, and normal S2. The peripheral pulses were of high volume. On CNS examination, Glasgow Coma Scale (GCS) was 15/15 and higher mental functions were intact. Motor, sensory, and cerebellar examinations were normal. Signs of meningeal irritation were absent and fundoscopy revealed no abnormalities. On pulmonary examination, there were no intercostal or substernal recessions. No use of accessory muscles was observed and the chest expansion was bilaterally symmetric. Normal vesicular breathing was present on the left side and upper and middle lobes on the right side. Crepitations were audible in the right lower lobe posteriorly, which did not improve or change character during coughing. On abdominal examination, pulsations were observed in the epigastric region. No scars, striations, or dilated veins were visible. The liver was palpable one finger below the costal margin with a soft and smooth lower border and a span of 14 cm. The spleen was mildly enlarged and palpable one finger below the costal margin. No fluid thrill or shifting dullness was present.

Laboratory investigations and biochemical and hematological tests, including complete blood count (CBC), electrolytes, serum urea, serum creatinine, and liver function tests (LFT), were all within normal parameters except for high leukocyte count (17000/μL). Viral markers including hepatitis B surface antigen and hepatitis C antibody were non-reactive. Prothrombin time (PT) was 20.6 seconds, activated partial thromboplastin time (aPTT) was 38.8 seconds, and the international normalized ratio (INR) was 1.9. The arterial blood gas showed a pH of 7.45, partial pressure of oxygen in arterial blood (PaO2) of 53.2 mmHg, partial pressure of arterial carbon dioxide (PaCO2) of 33.1 mmHg, HCO3 of 22 mEq/L, and oxygen saturation of 88%. Blood and urine cultures were taken, which turned out to be unremarkable. Table 1 lists the values of the laboratory tests.

**Table 1:** Laboratory values PT: prothrombin time; aPTT: activated partial thromboplastin time; INR: international normalized ratio; PCO2: partial pressure of carbon dioxide; PO2: partial pressure of oxygen

Laboratory	Value	Normal Value
WBC	17000/μL	4.5 to 11.0 × 10^9^/L
Hep B Surface Antigen	Negative	Negative
Hep C Antibody	Negative	Negative
PT	20.6 seconds	10 to 13 seconds
aPTT	38.8 seconds	21 to 35 seconds
INR	1.9	<1.1
pH	7.45	7.35 to 7.45
PCO2	33.1 mmHg	35 to 45 mmHg
P02	53.2 mmHg	75mmHg-100mmHg
HCO3	22 mEq/L	22-29 mEq/L

**Figure 1:**
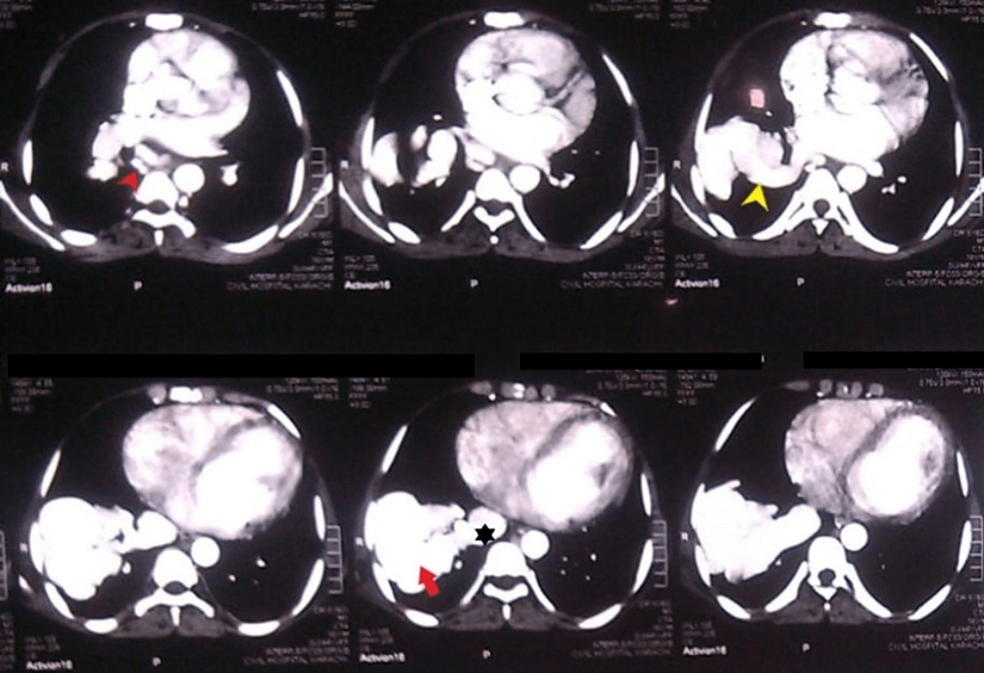
Contrast-enhanced computed tomography (CT) angiogram of the chest It demonstrated a serpiginous hypervascular mass in the lower lobe of the right lung measuring 7.0 x 8.1 x 5.3 cm.

Taking into account the alarming history of progressive dyspnea in a young patient and the history of HHT, contrast-enhanced computed tomography (CT) angiogram of the chest (Figure 1), which demonstrated a serpiginous hypervascular mass in the lower lobe of the right lung measuring 7.0 x 8.1 x 5.3 cm.

**Figure 2:**
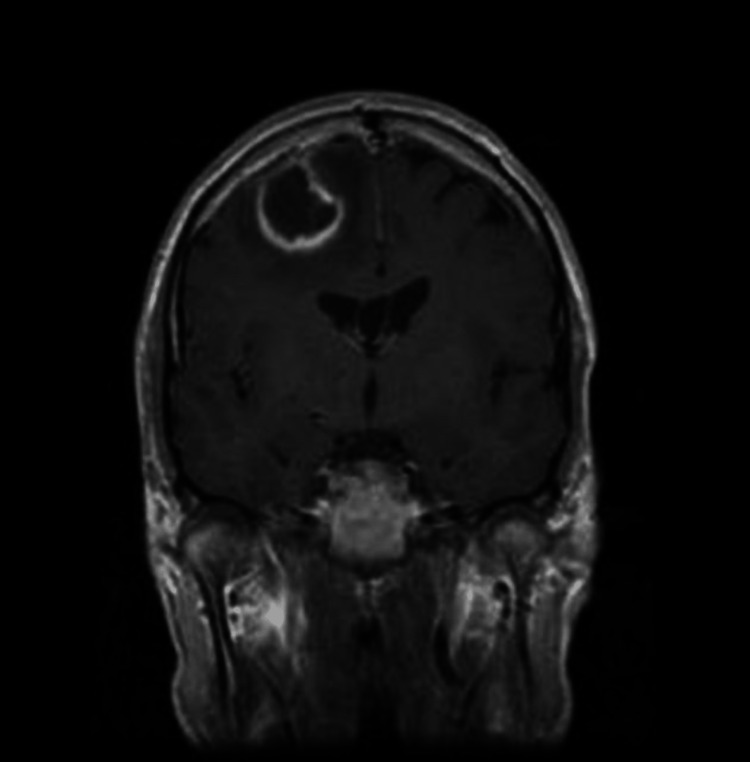
Magnetic resonance imaging (MRI) of the brain, a T1-weighted scan It reveals a 5.2 x 2.7 x 4.7 cm brain abscess in the temporoparietal region.

 It displayed communication with dilated right pulmonary artery, right inferior pulmonary vein, and multiple collaterals in the mediastinum around azygous and hemizygous veins, giving a picture of arteriovenous malformations. A decision of embolization was made to prevent any event of hemoptysis; however, the patient did not agree to undergo the procedure. During the second day of the hospital stay, the patient developed an episode of tonic-clonic seizure, which resolved within 15 seconds. Magnetic resonance imaging (MRI) of the brain was sought. A T1-weighted scan revealed a 5.2 x 2.7 x 4.7 cm brain abscess in the temporoparietal region (Figure 2).

There was no enhancement in the central portion, but the surrounding rim exhibited intense enhancement. Surrounding edema and mass effect were present. A diagnosis of cerebral abscess as a possible complication of HHT was made. A trial treatment regimen comprising ceftriaxone, vancomycin, and a corticosteroid was initiated and continued for four weeks. The patient showed remarkable improvement in his symptoms and was discharged after four weeks. The patient was completely asymptomatic at two months of follow-up. However, he was advised to follow up every three to six months for regular monitoring of bleeding based on his diagnosis of HHT.

## Discussion

Hereditary hemorrhagic telangiectasia (HHT) is an autosomal dominant vascular disorder that manifests as epistaxis, cutaneous telangiectasia, and visceral arteriovenous malformations (AVMs) as apparent in our patient. These arteriovenous malformations can arise in the pulmonary, portosystemic, cerebral, or spinal cord circulations and hence explain the neurological manifestations of HHT [5]. These manifestations can be diagnosed as transient ischemic attacks (TIA), intracerebral and subarachnoid bleeds, stroke, brain abscesses, or seizures, with the net result of paradoxical emboli passing between the right-to-left shunt in one-third of patients with pulmonary AVM that can lead to brain abscesses [4,6]. Moradi et al. proclaim that the parietal lobe is the most common site of abscesses, as is evident in our patient with involvement of the temporoparietal region [7]. The review of the literature tells us that the incidence of brain abscess in HHT is reported to be around 1.3/100,000 with high mortality in the range of 30-70%, but the introduction of CT and magnetic resonance imaging (MRI), along with more efficient antibiotics, has drastically decreased this number to around 10-15% [8]. Current medical literature proposes three mechanisms to explain the development of abscesses in HHT. First, the presence of an infarct allows bacteria to flourish. Second, septic emboli produce an infarct and a source of bacterial growth. Last, cerebral hypoxia and polycythemia, together create a favorable environment for bacteria to grow [5]. According to Han, a patient with a brain abscess presents with signs of the classic triad (cyanosis, polycythemia, and clubbing of the finger and toes) [9]. Our patient had fevers and seizures more classic of brain abscess but also had symptoms of cyanosis of the fingers and clubbing as seen on general physical examination. Like other cases of brain abscess, the treatment of HHT-associated brain abscesses also requires a combined medical and surgical approach [6]. Surgical drainage is performed for lesions greater than 2.5 cm in diameter, CT or magnetic resonance is used for progress monitoring, and six to eight weeks of IV antibiotic therapy is required [10]. Nonoperative treatment of brain abscesses has also been successful in some cases [6]. Our patient also improved only with antibiotics and no surgical approach was needed, which is quite a rare finding in HHT-associated brain abscesses.

## Conclusions

As our study reports, when a patient with a previous diagnosis of HHT presents with acute neurological symptoms, such as seizures, clinicians must maintain a high degree of suspicion and perform all relevant imaging modalities at an early stage to offer a better prognosis and significantly reduce the mortality rate due to patients with HHT that carry a significant risk of developing other neurological complications such as brain abscesses.

## References

[1] Lacombe P, Lacout A, Marcy PY (2013). Diagnosis and treatment of pulmonary arteriovenous malformations in hereditary hemorrhagic telangiectasia: An overview. Diagn Interv Imaging.

[2] van Gent MW, Velthuis S, Post MC, Snijder RJ, Westermann CJ, Letteboer TG, Mager JJ (2013). Hereditary hemorrhagic telangiectasia: how accurate are the clinical criteria?. Am J Med Genet A.

[3] McDonald J, Bayrak-Toydemir P, Pyeritz RE (2011). Hereditary hemorrhagic telangiectasia: an overview of diagnosis, management, and pathogenesis. Genet Med.

[4] Saboo SS, Chamarthy M, Bhalla S (2018). Pulmonary arteriovenous malformations: diagnosis. Cardiovasc Diagn Ther.

[5] Mathis S, Dupuis-Girod S, Plauchu H (2012). Cerebral abscesses in hereditary haemorrhagic telangiectasia: a clinical and microbiological evaluation. Clin Neurol Neurosurg.

[6] Kikuchi Kenji, Kowada Masayoshi, Sasajima Hiroyasu (1994). Vascular malformations of the brain in hereditary hemorrhagic telangiectasia (Rendu-Osler-Weber disease). Surg Neurol.

[7] Moradi M, Adeli M (2014). Brain abscess as the first manifestation of pulmonary arteriovenous malformation: a case report. Adv Biomed Res.

[8] Härkönen M (1981). Hereditary hemorrhagic telangiectasia (Osler-Weber-Rendu disease) complicated by pulmonary arteriovenous fistula and brain abscess. Acta Med Scand.

[9] Han S, Lim DJ, Chung YG (2002). The multiple brain abscesses associated with congenital pulmonary arteriovenous malformations: a case report. J Korean Med Sci.

[10] Mathisen GE, Johnson JP (1997). Brain abscess. Clin Infect Dis.

